# A Surgical Treatment for Adult Muscular Torticollis

**DOI:** 10.1155/2013/965693

**Published:** 2013-05-16

**Authors:** Song Ho Chang, Seiji Ohtori, Akihiko Okawa, Koui Kawamura, Hiroshi Saiki, Izumi Nakada, Takashi Shimada, Junichi Nakamura, Kazuhisa Takahashi, Hiroshi Sugiyama

**Affiliations:** ^1^Department of Orthopaedic Surgery, Graduate School of Medicine, Tokyo University, Bunkyo-ku, Tokyo 113-8655, Japan; ^2^Department of Orthopaedic Surgery, Asahi General Hospital, Asahi, Chiba 289-2511, Japan; ^3^Department of Orthopaedic Surgery, Graduate School of Medicine, Chiba University, 1-8-1 Inohana, Chuo-ku, Chiba 260-8670, Japan

## Abstract

Adult presentation of neglected congenital muscular torticollis (CMT) is rare. Therefore, efficacy of surgical treatment for adult CMT is unclear. We experienced a case of neglected CMT in a 28-year-old male patient and report the surgical result here. We conducted unipolar resection at the distal end of the sternocleidomastoid muscle (SCM). After surgery, the range of neck movement and head tilt improved, and his appearance was cosmetically improved despite the long-standing nature of the deformity. We concluded that surgical management of adult patients with neglected congenital muscular torticollis may be a treatment option.

## 1. Introduction

Congenital muscular torticollis (CMT) is the third most common congenital musculoskeletal anomaly after dislocation of the hip and clubfoot [1]. The term congenital muscular torticollis (CMT) refers to a neck deformity that primarily involves shortening of the sternocleidomastoid muscle that leads the head to turn toward the affected side and the chin to point to the opposite side [2]. When diagnosed early, it is obvious that it can be managed with good or excellent results [2]. Ling et al. have stated that the optimal time for surgery is between 1 and 4 years [3, 4]. This is based on the finding that most children treated before the age of 1 year respond well to conservative treatment [3, 4]. However, there are few reports that indicate the efficacy of surgery for neglected cases in adults [5, 6]. In the current report, we experienced a case of neglected muscular torticollis in a 28-year-old patient. We report the efficacy of unipolar tenotomy of the sternocleidomastoid muscle (SCM) for this adult patient.

## 2. Case Presentation

This report was approved by the patient after informed consent. A 28-year-old male patient had a diagnosis of CMT at birth. However, his treatment was neglected until he began to care about his cosmetic appearance. He came to our clinic when he was 28 years of age, and he hoped for surgical treatment to improve his cosmetic problem. He had no clinical past history and no family history.

**Figure 1:**
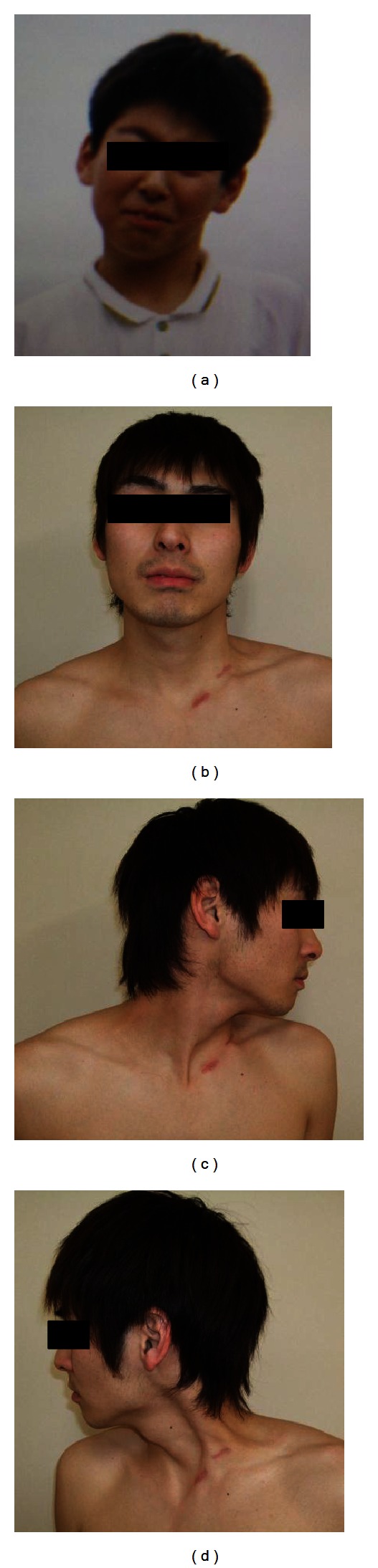
A 28-year-old man with muscular torticollis. (a) Preoperative photograph showing severe head tilt. (b) Postoperative photograph taken 12 months after surgical release showing an excellent cosmetic result. (c and d) Postoperative photographs taken 12 months after surgical release showing range of motion in the neck.

He showed slight facial asymmetry. He had a 7° right side rotational deficit and 35° lateral flexion deficit before surgery. We could palpate a hard mass in the SCM in his left neck. We planned resection of the SCM for the patient. At first, unipolar resection at the distal end of SCM was performed. Rotation and lateral flexion at the left side improved, and tension of the SCM disappeared during surgery; we did not add resection at the proximal end of the SCM. There was no significant complication after surgery. We programmed active, passive rotation and flexion rehabilitation from the next day. Twelve months after the surgery his neck rotational deficit improved to 2° and lateral flexion deficit to 3°. There was no complication or recurrence during the 12 months of follow-up (Figure 1).

A scoring system proposed by Lee et al., which included function and cosmetic results, has been used for assessing the surgical outcome [7]. The neck movement and lateral band were compared with the uninvolved side, and the head tilt and operative scar were evaluated by clinical observation and a questionnaire (Table 1; modified from [7]). The total possible score is 18 points. For both pre- and postoperative assessment, the following system is used: 17-18 points, excellent result; 15-16 points, good result; 13-14 points, fair result; and <12 points, poor result. The patient's preoperative score was 6. The postoperative score was 15 (good result), and the patient's satisfaction was excellent.

## 3. Discussion

In the current study, we experienced a case of neglected CMT in a 28-year-old male patient. We performed unipolar resection at the distal end of the SCM. The range of neck movement and head tilt improved, and his appearance was cosmetically improved despite the long-standing nature of the deformity. We can recommend surgical management of adult patients with neglected congenital muscular torticollis.

Most cases of CMT resolve completely either spontaneously within months after birth or with conservative measures initiated early, such as gentle controlled passive manual stretching exercises on the affected side. Sönmez et al. found that 95% of patients diagnosed and treated effectively before the age of one year did not need surgical treatment [8]. In patients seen later, surgical intervention should be considered as the treatment of choice in order to avoid further irreversible changes. Surgery is also recommended in patients with residual head tilt, passive rotation deficit, or lateral bending of more than 15° at the age of 6 months [9].

The timing of surgery is controversial. Canale et al. reported that full recovery of facial asymmetry after the age of 4 is difficult to achieve [4]. Lee et al. [7], Minamitani et al. [10], and Chen and Ko [11] reported that late release of the sternocleidomastoid muscle for patients more than 6 years of age could yield acceptable results. On the other hand, there have been few reports of surgical treatment for adults (over 20 years old) [5, 6]. Eighteen adult and skeletally matured patients (18 to 32 (average 21.9) years) were surgically treated for neglected CMT and prospectively followed, and surgical results for most patients were excellent or good [5]. Twelve adult patients with neglected CMT (17 to 31 (average 24) years) were surgically treated and were followed up for a minimum of two years. Most patients showed excellent results in the range of neck movement and head tilt improved in all 12 patients and cosmesis improved in 11 [6]. In the current study, we also showed good surgical results in a patient with neglected CMT. In this regard, we recommend surgical treatment for adult patients.

A surgical method for adult patients with neglected CMT has been reported [5, 6]. Surgical bipolar sectioning of the SCM should be considered, even in adults with irreversible facial and skeletal deformities [5]. Moreover, surgical management of adult patients with neglected congenital muscular torticollis using a bipolar release of the SCM gives excellent results [6]. In the current study, we selected unipolar resection at the distal end of the SCM; rotation and lateral flection on the left side improved, and SCM tension disappeared during surgery. If we can recognize that SCM tension will decrease and limitation of the range of motion for the neck will improve, selection of unipolar resection is a surgical option for the adult patient with neglected CMT.

In conclusion, patients with CMT may benefit from surgical sectioning of the SCM even in adulthood. The surgery restores the range of neck motion, resolves the head tilt, and can therefore improve the patient's quality of life.

## Figures and Tables

**Table 1:** Scoring system for assessment of muscular torticollis (modified from [7]).

Points	Function			Cosmesis		
	Facial asymmetry	Neck movement	Head tilt	Scar	Loss of column	Lateral band
3	None	Full	None	Fine	None	None
2	Mild	Limited	Mild	Slight	Slight	Slight
1	Moderate	10°–25°	Moderate	Moderate	Obvious but cosmetically acceptable	Obvious but cosmetically acceptable
0	Severe	>25°	Severe	Unacceptable	Unacceptable	Unacceptable
